# Research on allergic rhinitis improvement in asthmatic children after dust mite exposure reduction: a randomized, double-blind, cross-placebo study protocol

**DOI:** 10.1186/s13063-020-04614-6

**Published:** 2020-07-29

**Authors:** Ming Chen, YuFen Wu, Shuhua Yuan, Jiande Chen, Luanluan Li, Jinhong Wu, Jing Zhang, Yong Yin

**Affiliations:** Department of Respiratory Medicine, Shanghai Children’s Medical Center affiliated to Shanghai Jiaotong University of Medicine, Shanghai, 200127 China

**Keywords:** Allergic rhinitis, Asthma, Dust mite

## Abstract

**Background:**

Allergic rhinitis (AR) in children is a major respiratory inflammatory disease with a high incidence that is increasing yearly. In China, 54.93% of children with asthma have AR, which often requires synchronous treatment. House dust mites (HDMs) are common allergens that often cause attacks of AR and asthma. Reducing allergen exposure is one of the most important measures to control and treat AR and asthma attacks. Hestelia Mite Bait, containing 0.1% emamectin, is a new tool for trapping and killing dust mites, reducing the number of dust mites on mattresses and thereby potentially reducing stimulation by allergens and ultimately improving asthma and rhinitis symptoms. This single-centre, randomized, double-blind, cross-placebo trial will explore the improvement in AR in asthmatic children after dust mite exposure reduction.

**Methods:**

We will recruit 60 children (aged 3–12 years) who have been diagnosed with AR and asthma and are allergic to dust mites as confirmed by a serum allergen test. Participants will randomly receive the Hestelia Mite Bait intervention for 8 weeks and the placebo intervention for 8 weeks. There will be a 4-week washout period between the two interventions. The primary outcome is the visual analogue scale (VAS) score of AR symptoms; the secondary outcomes include the Rhinitis Control Assessment Test (RCAT) score, Rhinoconjunctivitis Quality of Life Questionnaire (RQLQ) score, changes in the dust mite level, drug usage for asthma and AR, Asthma Control Questionnaire-5 (ACQ-5) score, and frequencies of acute asthma attacks, emergency visits, and hospitalizations.

**Discussion:**

This study aims to scientifically and objectively evaluate the effects of mite bait on rhinitis and asthma improvement after dust mite exposure reduction and provides a convenient means for future prevention and treatment of allergic diseases involving the airways in children.

**Trial registration:**

www.chictr.org.cn ChiCTR1900024688. Registered on July 21, 2019

## Introduction

### Background and rationale

Allergic rhinitis (AR) is a non-infectious disease of the nasal mucosa mediated by immunoglobulin E (IgE) after exposure to allergens. AR is a common allergic disease in children. The incidence of AR has increased to 10–30% in adults and to 40% in children [1]. AR manifests as sneezing, rhinorrhoea, sinus itch, and other symptoms, which have an adverse effect on quality of life, leading to disordered sleep breathing and increased incidence of attention deficit disorder [2, 3].

AR often coexists with organ-complicated allergic diseases, among which asthma is one of the most common conditions. In a Chinese epidemiological study, the incidence of asthma in children with AR was 35.01%, and the prevalence of AR in children with asthma was 54.93% [4]. AR and asthma have common pathophysiological elements. The immunopathologies of AR and asthma are similar in the cellular influx of eosinophils, mast cells, and T helper type 2 (Th2) cells. A similar array of mediators can be found in the lavage fluid of patients with AR and asthma [5]. Therefore, AR and asthma are proposed as “the same respiratory tract, the same disease”. In the presence of asthma, treatment of AR is often overlooked, and poor control of AR has been shown to be one of the causes of refractory and recurrent asthma [6–8]. Control of AR reduces the incidence of asthma attacks and associated hospitalizations [9].

Dust mites are a common trigger for AR [10]. The prevalence of house dust mites (HDMs) in AR patients in southern China is 95% [11]. Avoiding allergen exposure is an important measure to treat AR. Currently, reduced HDM exposure measures include keeping room humidity below 50%, wrapping mattresses and pillows with impervious covers, regularly cleaning bedding with hot water, removing carpets and plush toys, and regularly using high-efficiency particulate air filters and acaricides. A systematic review of randomized controlled trials was conducted in which HDM control measures were evaluated compared to placebo or other HDM avoidance measures in patients with clinically proven AR. In this review, seven of the nine trials reported that the interventions studied resulted in significant reductions in HDM load compared with the control. However, of the interventions studied to date, acaricides appear to be the most promising [12]. Hestelia Mite Bait, containing 0.1% emamectin, attracts dust mites into a mite trapping bag through the combined action of an oligomeric aromatic factor and dust mite atopic agents; then, it kills the dust mites, thereby achieving the goal of reducing allergens. Whether this new, safe, and effective tool to trap and kill dust mites can improve rhinitis and asthma symptoms has not been verified.

### Objectives

The purpose of this study is to evaluate the improvement in rhinitis and asthma symptoms after reduction of dust mite exposure with the acaricide Hestelia Mite Bait through a randomized, double-blind, cross-placebo clinical trial.

### Trial design

This is a randomized, double-blind, cross-placebo clinical trial to determine the improvement in rhinitis and asthma symptoms after dust mite exposure reduction. Participants will be randomly divided into 2 groups (group 1 and group 2). From the first day to the 8th week, the patients will apply mite baits or the placebo packages and complete questionnaires three times (V1–V3, V1 = the first day after enrolment; V2 = the fourth week plus or minus 3 days after enrolment; V3 = the eighth week plus or minus 3 days after enrolment). House sampling will be conducted twice (V1 and V3) during this period. After 4 weeks of washout, day 85 marks the beginning of the second phase of treatment.

During this period, the patients will be crossed to accept different interventions for 8 weeks. Questionnaires will be completed three times again (V4–V6, V4 = the twelfth week plus or minus 3 days after enrolment, V5 = the sixteenth week plus or minus 3 days after enrolment, V6 = the twentieth week plus or minus 3 days after enrolment), and house sampling (V4 and V6) will be completed twice again. Finally, we will conduct the questionnaire survey 4 weeks after the end of the intervention (V7, V7 = the twenty-fourth week plus or minus 3 days after enrolment). Throughout the study, the children will receive standard treatments in accordance with asthma and AR guidelines [13]. The efficacy of mite bait in reducing AR and asthma symptoms will be assessed using a visual analogue score (VAS) for rhinitis symptoms, Rhinitis Control Assessment Test (RCAT) score, Rhinoconjunctivitis Quality of Life Questionnaire (RQLQ) score, changes in the level of dust mites, drug usage for asthma and AR, Asthma Control Questionnaire-5 (ACQ-5), and frequencies of acute asthma attacks, emergency visits, and hospitalizations.

## Methods: participants, interventions, and outcomes

### Study setting

The target population for this trial will be recruited from the respiratory clinic of Shanghai Children’s Medical Center, a tertiary first-class paediatric hospital with a large number of outpatients with asthma and rhinitis and a professional respiratory medical team.

### Eligibility criteria

#### Participants

Inclusion criteria

1. Aged 3–12 years, male or female.
2. Children diagnosed with AR in accordance with the 2019 guidelines for diagnosis and treatment of AR [13]. At the same time, the diagnosis must conform to the diagnostic criteria for childhood asthma formulated by the National Children’s Asthma Prevention and Treatment Cooperation Group in 2016 [﻿14﻿].
3. Maintained use of guide-based rhinitis and asthma control drugs for the previous 1 month. Inhaled drugs, such as budesonide suspension, fluticasone aerosol, salmeterol dry powder inhalation, and budesonide formoterol dry powder inhalation, could be used to control the condition according to age characteristics.
4. Performance of the serum-specific allergen test, with the level of dust mite allergen sIgE > 0.35 IU/mL considered positive.
5. Written informed consent provided by the guardians of all subjects (approved by the Ethics Committee of Shanghai Children’s Medical Center affiliated with Shanghai Jiao Tong University School of Medicine).
6. Agreement to collect dust mites from indoor mattresses.

Exclusion criteria

1. Basic diseases, such as congenital heart disease, immune deficiency, gastroesophageal reflux, bronchopulmonary dysplasia, and obliterative bronchiolitis.
2. Inability to sleep in a separate bed.
3. Participation in other clinical studies within the past 3 months.

### Process of obtaining informed consent

Hui Li, a staff member hired by the trial sponsor, will post recruitment information on the WeChat official account “Respiratory Angel” of the Department of Respiratory Medicine of the Shanghai Children’s Medical Center and collect the information of participants through Wenjuanxin, a platform providing functions equivalent to Amazon Mechanical Turk. Finally, Hui Li will contact the guardians of qualified patients through WeChat and receive written informed consent.

### Additional consent provisions for collection and use of participant data and biological specimens

On the consent form, participants will be asked if they agree to the use of their data should they choose to withdraw from the trial. Participants will also be asked for permission for the research team to share relevant data with people from the universities participating in the research or from regulatory authorities, when relevant. This trial does not involve the collection of biological specimens for storage. The form used to obtain informed consent is available from the corresponding author on request.

## Interventions

### Explanation for the choice of comparators

We will compare Hestelia Mite Bait containing 0.1% emamectin with a placebo that has a consistent appearance and odour but no acaricidal effect to investigate the change in dust mite exposure and the improvement in asthma and rhinitis symptoms after indoor use of the mite bait.

### Intervention description

**Fig. 1:**
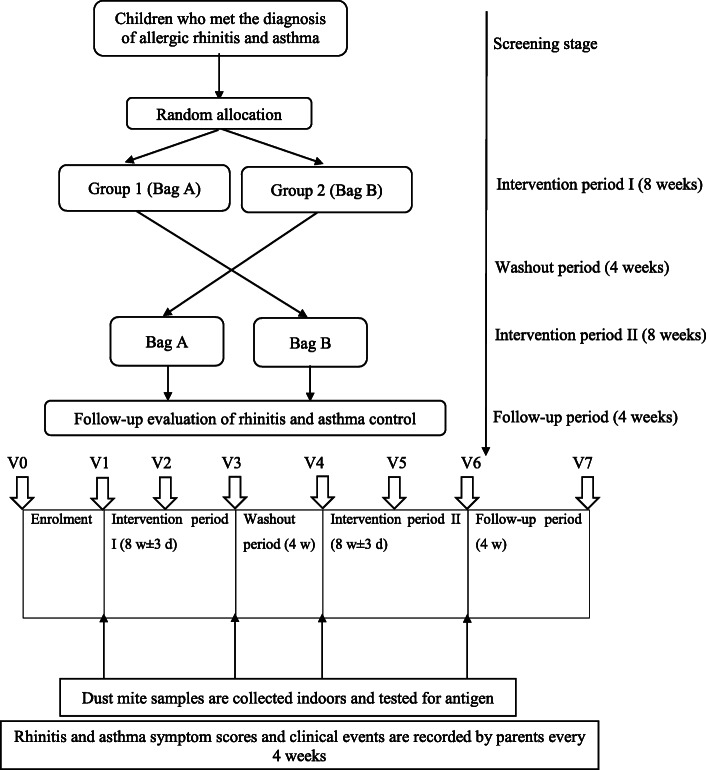
Study flow chart

After recruitment (V0), baseline medical characteristics (name, age, sex, diagnosis of asthma and rhinitis and the allergen test report) will be collected. The subjects will be randomly divided into 2 groups. The children randomly assigned to group 1 will be placed with package A for an 8-week intervention. All HDM species reach adulthood within 3 to 4 weeks. Once mature, adult mites have a life expectancy of between 4 and 6 weeks [15]. To avoid an impact of the first intervention on the subsequent intervention, we will establish a 4-week washout period according to the growth cycle of the dust mites. After the 4-week washout period, package B will be placed for an 8-week intervention. In group 2, package B will be placed for an 8-week intervention, followed by a 4-week washout period and then package A will be placed for an 8-week intervention. Each child will undergo a total intervention period of 16 weeks, a washout period of 4 weeks, and a follow-up period of 4 weeks after the end of the second intervention. A flow chart of the study is shown in Fig. 1. At V1, V3, V4, and V6, the staff will collect indoor samples from mattresses using a glass fibre membrane, mite-clearing vacuum cleaner. The components of dust mite antigens in the collected samples will be detected via enzyme-linked immunosorbent assays. Parents will be asked to evaluate the AR and asthma symptom scores and clinical event records at V1–V7 (see the outcome indicators for details), as shown in Table 1.

**Table 1:**
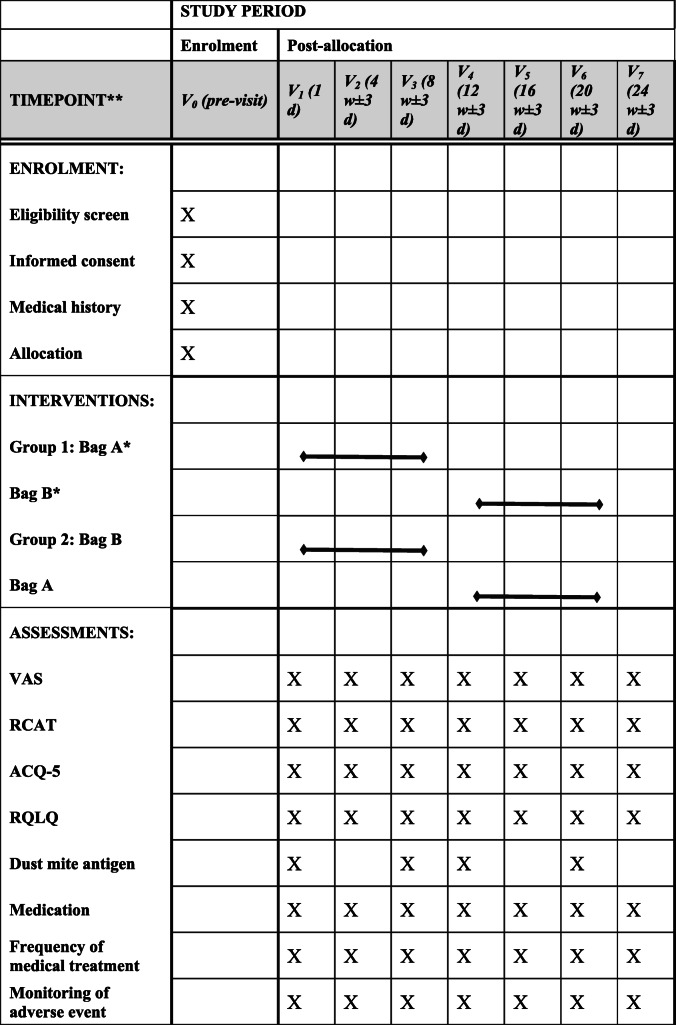
Study schedule

*Bag A and bag B are indistinguishable from each other in terms of packaging and smell, and whether they contain placebo or mite bait will be known only to the pharmacist

### Criteria for discontinuing or modifying allocated interventions

If the research physician feels that it is not in the child’s best interest to continue participating in the study (e.g. if there is an allergic reaction to the mite bait or placebo used), he/she may decide whether to withdraw the child from the study at any time. If a subject’s parents fail to complete the questionnaire after 3 reminders or fail to cooperate with the indoor sample collections, the subject will be considered poorly compliant and will be excluded from the study.

### Strategies to improve adherence to interventions

First, we will explain our study to each child’s parents as follows: the enrolled children will receive regular follow-up, a questionnaire evaluation, and standardized treatment by respiratory specialists at Shanghai Children’s Medical Center. The detection of the HDM antigen concentration in the bedroom mattress is free of charge, and the mite bait and placebo package are safe. This is the only hygienic product for dust mite removal certified by the Ministry of Agriculture in China and has a product registration certificate (registration certificate number: WP20180004). During the study, professional staff will enter the room four times to clean the mattress using a mite-removing vacuum cleaner and collect the attractors. The entire process requires the cooperation of family members, and parents need to complete the questionnaires within 10–20 min.

Second, during the implementation process, we will regularly remind parents to fill out the questionnaire via WeChat. The research team will provide help and detailed answers to parents’ questions during the trial.

### Relevant concomitant care permitted or prohibited during the trial

The original asthma medication can be maintained during the trial. When acute asthma attack symptoms occur, β2 agonists will be used to relieve bronchospasm, and depending on the clinical severity, oral or intravenous corticosteroids will be used until the symptoms are relieved. However, during the test, the children will not be allowed to go outside for long periods of time and must remain indoors in a room with mite bait or placebo at night when they sleep.

### Provisions for post-trial care

Patients with adverse reactions that occur in the context of the study will be followed up by the investigator and receive help from our professional medical team until the problem is resolved. After the entire test, each family will get the bait mite for free.

### Outcomes

#### Primary outcome

- VAS for clinical symptoms of AR
  ○ In 1988, Linder first applied the VAS to assessment of AR symptoms, demonstrating its sensitivity and specificity [16]. Patients will be scored using the VAS for symptoms occurring during the previous week, including sneezing, rhinorrhoea, nasal itching, nasal congestion, itchy eyes, teary eyes, foreign body sensation, and red eyes, for a total of eight symptoms. The VAS is a 10-cm line bounded on one end with a score of 0 and on the other end with a score of 10 that is used to show the severity of a patient’s symptoms (“0” for no symptoms and “10” representing the most severe symptoms). Patients will be directed to mark the symptom scores on the scale according to their perception of their symptoms.

#### Secondary outcomes

- Change in the RCAT score
  ○ The RCAT demonstrated adequate reliability, validity, and responsiveness and was deemed acceptable and appropriate by the patients. This tool can facilitate detection of AR symptom-control problems, and its brevity supports its usefulness in clinical care. The RCAT consists of 6 items: nasal congestion, sneezing, watery eyes, sleep problems caused by rhinitis, activity avoidance, and rhinitis symptom control. Responses are measured on a 5-point Likert-type scale. RCAT scores range from 6 to 30, with higher scores indicating better rhinitis control [﻿17﻿].
- Change in the ACQ-5 score
  ○ The ACQ-5 is a questionnaire composed of 5 simple multiple-choice questions that are scored on a scale. The results are obtained by adding the total points and averaging them. It is a rapid assessment tool and plays a significant role in evaluating the extent to which asthma is controlled. Each child will be asked to evaluate the level of asthma control in the previous week. The lower the score is, the better the control level.
- RQLQ for children with AR [﻿18﻿]
  ○ In this study, children with rhinitis during the previous 1–2 weeks will be evaluated on the basis of their symptoms, psychological status, mental status, social communication, and other aspects of 14 problems caused by rhinitis. The questionnaire is based on a scale according to the following point values: 0 points, normal; 1 point, slight; 2 points, mild; 3 points, serious; and 4 points, very serious. The higher the score is, the more severe the effect of rhinitis on the quality of life.
- Changes in levels of dust mite antigen in children’s beds
  ○ Three sampling points will be randomly selected for each mattress, and each sampling point will have a range of 30 cm^2^. Each sampling point will be repeatedly vacuumed 10 times using a glass fibre membrane, mite-clearing vacuum cleaner (the bed area will be recorded at the same time). Dust on the glass fibre membrane in the vacuum cleaner will be put into a plastic bag and stored at − 20 °C (killing the dust mites). The samples from each family will be weighed, and then, allergens will be extracted. ELISAs (Indoor Biotechnologies, Charlottesville, VA, USA) will be used to detect the dust mite antigens Der p2 (*Dermatophagoides pteronyssinus*) and Der f2 (*D. farinae*) in the extracted solution.
- Use of medicines for AR and asthma in children
  ○ The percentages of the children who frequently used anti-asthma drugs for control of rhinitis during the previous 4 weeks were as follows: no use ever, 0%; a total of 1 week of use, 25%; and use every day, 100%. Specific drugs include physiological saline, nasal spray hormone (Mometasone Furoate Aqueous Nasal Spray, Fluticasone Propionate Nasal Spray, Budesonide Nasal Spray, etc.), oral allergy drugs (cetirizine, loratadine, levocetirizine, desloratadine, etc.), Sinupret Drops, montelukast, traditional Chinese medicine (Tongqiao Biyan Granule, Biyuanshu Oral Liquid, Xinqin Granule), nasal allergy medications (levocabastine, azelastine hydrochloride), desensitization treatment (Dermatophagoides Farinae Drops), and inhaled hormones to control asthma (Budesonide Suspension For Inhalation, Seretide, Flixonase, Symbicort Turbuhaler).
- Number of asthma attacks, emergency visits, frequency of hospitalization

### Sample size

Based on the assumption that a reduction of 25% in VAS scores would be of clinical significance, enrolment of 44 patients in each group is required at the 5% significance level (two-tailed), with a power of 90% to detect differences between the two groups [19]. Considering a 10% possible dropout rate, at least 49 people need to be enrolled in each group.

In this study, we will recruit 60 people for a placebo-controlled, double-blind, crossover trial. After crossover, both the placebo and experimental groups will each be increased to 60 people.

### Recruitment

Recruitment information will be posted on the WeChat official account “Respiratory Angel” of the Department of Respiratory Medicine of the Shanghai Children’s Medical Center.

## Assignment of interventions: allocation

### Sequence generation

Random sequences will be generated using a random number table.

### Concealment mechanism

Random sequences will be successively assigned to the subjects according to the enrolment order.

Odd-numbered subjects will be entered into group 1, and even-numbered subjects will be entered into group 2. Three copies of the generated distribution sequence table will be distributed among the designer, pharmacist, and statistician. Each copy will be sealed in an opaque envelope and kept under lock.

### Implementation

A special person who is not involved in the subsequent grouping and intervention will be responsible for enrolling the test subjects according to the selection and exclusion criteria. A random sequence will be generated by the statistician. The test designer has decided that odd-numbered subjects will be entered into group 1, and even-numbered subjects will be entered into group 2.

## Assignment of interventions: blinding

### Who will be blinded

The placebo used in the study looks and smells indistinguishable from mite bait and will be labelled either A or B. The identity of tag A or tag B will be known only to the pharmacists and unknown to the subjects and researchers.

### Procedure for unblinding if needed

When the trial is over, the number of each subject and the treatment plan received need to be checked, and the sealed distribution sequence needs to be decrypted. When unblinding, the intervention measures recorded in the assigned serial number will be checked with the drug delivery record sheet, and the results data will be classified as the test group or the control group for analysis.

## Data collection and management

Baseline data and questions related to the outcome indicators will be designed into questionnaires at https://www.wjx.cn/ and regularly assigned to parents to complete via WeChat. The parents or guardians who will complete the questionnaires are the individuals mainly responsible for the daily life of the child. During the follow-up period, once the questionnaire is completed and submitted, the contents of the questionnaire will not be available for any modifications. In addition, a primary staff member will check whether the parents or guardians completed the questionnaire properly. The staff member will not know the parent grouping or intervention. At the conclusion of the entire experiment, all data will be exported to EXCEL (Microsoft Corporation, Redmond, WA) and analysed using SPSS 20.0 software (SPSS Inc., Chicago, IL).

## Statistical methods

### Statistical methods for primary and secondary outcomes

SPSS 20.0 software will be used to analyse the experimental data. Descriptive statistics will be used for the following data analyses: categorical variables, namely, RCAT scores, ACQ-5 scores, drug usage for asthma and AR, and frequencies of acute asthma attacks, emergency visits, and hospitalizations via frequency tables (i.e. number of evaluable subjects, frequency and percentage for categorical values). The mean ± SD, median, minimum, and maximum will be used as continuous variables, namely, the VAS scores and RQLQ scores. One-way analysis of variance and a two-sample *t* test will be adopted for normally distributed data, and a non-parametric rank-sum test will be adopted for non-normally distributed data. Fisher’s exact and chi-square tests will be used to compare classified data, and *P* < 0.05 will be considered statistically significant.

### Interim analyses

At the end of the first phase of the test (V3), an interim analysis of the data from the previous stage will be conducted by the biostatistician of the data monitoring committee, who will primarily analyse the size of the estimated effect in advance and the incidence of adverse events. If there is a lack of treatment effect or the incidence of adverse events is high, early termination of the trial will be considered. These results will be reported back to the trial sponsor, and he or she will decide whether to terminate the experiment or not.

### Methods for additional analyses

When evaluating factors affecting rhinitis and asthma control, multiple linear regression or logistic regression will be used.

### Analysis methods to account for protocol non-adherence and statistical methods to be applied for missing data

For subjects that are randomized to the intervention but do not adhere to the intervention, if they completed the first phase of the trial before the crossover, their data will be retained, and the shortest distance filling method or multiple filling method will be used to assess the missing data; otherwise, the observed samples will be deleted.

### Access to the full protocol, participant-level data, and statistical codes

The datasets analysed during the current study are available from the corresponding author on reasonable request.

## Oversight and monitoring

### Composition of the coordinating centre and Trial Steering Committee

In this trial, the sponsor hires staff member Hui Li to publish recruitment information through WeChat and collect basic information of the participants. The information will be shared with Yufen Wu to select qualified research subjects. After confirming eligibility, Hui Li will contact the child’s guardian and acquire written informed consent. Throughout the trial, two professional staff members will be responsible for collecting indoor samples and replacing mite bait or placebo. At the same time, one staff member will be responsible for regularly providing electronic questionnaires to the enrolled subjects to follow-up the symptoms of the children and summarize the adverse events during the trial. Ming Chen will evaluate the reliability of each uploaded follow-up questionnaire. During the trial, the Trial Steering Committee, composed of Lirong Jiang and Yijiong Ren, will review the progress and safety of the trial.

### Composition of the data monitoring committee

The data monitoring committee (DMC) includes a biostatistician, a respiratory professional physician, and a patient advocate to advise the sponsors and principal investigators regarding the continuing safety of study patients and the scientific merit of the study. The DMC is independent of the sponsor and competing interests. The DMC is responsible for monitoring the recruitment of participants, compliance with the protocol and data quality, serious adverse events, and other safety questions.

### Adverse event reporting and harms

During the trial, we will collect and record the occurrence of adverse events, describe the date of onset and date of resolution, evaluate event severity, investigate potential causal relationships with the intervention or with other suspect drugs, and assess the potential effect of the event on the final outcome, which will be reported to the DMC and ethics committee.

The mite bait will be placed under the mattress and will not be in direct contact with the child. However, the acute oral, transdermal, and inhalation toxicity of the 0.1% emamectin in the mite bait is considered to be slight, and thus, the mite bait needs to be secure. Some adverse events that may occur with emamectin include skin and eye irritation, nausea, vomiting, headache, dizziness, fatigue, chest tightness, excessive sweating, salivation, blurred vision, convulsions, and tachycardia or bradycardia.

Severity should be defined according to the following criteria:

- Mild: Awareness of signs or symptoms, but easily tolerated
- Moderate: Sufficient discomfort to cause interference with normal daily activities
- Severe: Inability to perform normal daily activities
- Life threatening: Immediate risk of death from the reaction as it occurred

All adverse events will be tracked until the incident is resolved or the study is completed.

### Frequency and auditing of trial conduct

The Project Management Group will meet once a month to review the trial. The Trial Steering Group and the independent Data Monitoring and Ethics Committee will meet to review conduct throughout the trial period 12 months after the trial is approved.

## Dissemination plans

The investigators and sponsor will communicate trial results to participants, healthcare professionals, and the public via the WeChat official account “Respiratory Angel” and publication of the study.

## Discussion

In recent years, the prevalence of allergic diseases has been increasing. In the past 20 years, China has conducted three national epidemiological surveys on asthma in children. The results show that the average prevalence of asthma among children aged 0–14 years was 1.08% in 1990. In 2000, this number increased to 1.97%. In 2010, when 400,000 children were surveyed, the prevalence was 3.01%, up approximately 50% from 2000 [﻿20﻿]. Zhao Jing et al. adopted a multistage sampling method to conduct epidemiological investigations of children with AR in Beijing, Chongqing, and Guangzhou and found that the incidence rates of AR were 14.46%, 20.42%, and 7.83%, respectively. At the same time, it was found that the incidence level of AR in China was gradually increasing, and the gap with developed countries had narrowed [﻿21﻿]. Consequently, allergic diseases are increasingly affecting people’s health and quality of life [1].

Taking asthma as an example, the causes of allergic diseases are mostly related to indoor allergens, such as dust mites, moulds, and animal dander, among which dust mites are the most frequently involved [﻿22﻿]. A longitudinal population-based study, which included 29 centres (14 countries) mostly in western Europe, showed that AR related to dust mite allergies was associated with an increased risk of asthma independent of other allergens [﻿23﻿].

There is still no ideal treatment for diseases caused by dust mite allergy. In clinical practice, dust mite antigen extract infusion or drug inhibition can be used to reduce the immune tolerance of the body and thereby alleviate or relieve symptoms. However, although patients with an allergic constitution can reduce their symptoms through treatment, this condition is difficult to completely cure [﻿24﻿, ﻿25﻿]. Therefore, compared with expensive and long-cycle treatment, controlling the number of dust mites in the house and reducing the exposure of patients to allergens is an inexpensive and easy method to promote.

In clinical practice, several common methods for physical mite removal exist [﻿26﻿], such as anti-mite bed covers and anti-mite vacuum cleaners, but these measures cannot significantly reduce the number of live dust mites and remove hidden allergens. Chemical control for different purposes can be divided into two types: acaricides and other types. Acaricides can quickly and effectively kill individual dust mites but cannot effectively remove dust mite carcasses, faeces, and other allergens, and as chemical agents, their safety cannot be guaranteed. Repellent only enables avoidance of dust mites but cannot isolate allergens and is characterized by a bad odour, a thick and oily texture, no resistance to sweat or washing, and other defects. However, the mite bait used in this study enables an oligomeric aromatic factor compound and dust mite atopic agents to work together to induce dust mites to enter the bag and ingest only the agents effective against dust mites until they die, thus effectively achieving the purpose of blocking allergens.

The main effective component of the mite bait, emamectin benzoate, is a low-toxicity insecticide and acaricide. Emamectin is a highly effective biological agent synthesized on the basis of avermectin and has the characteristics of super-high efficiency, low toxicity (nearly non-toxic), no residue, and no pollution. Compared with that of avermectin, the insecticidal activity of emamectin benzoate is improved by 1–3 orders of magnitude, and it has very high activity against the larvae of lepidoptera insects, mites, and many other injurious insects. Emamectin benzoate has both the gastric toxicity and action of a contact poison, with a good effect at a very low dose (0.084~2 g/ha) [﻿27﻿]. After testing, the 0.1% emamectin benzoate used in the mite bait has slight toxicity through the skin, mouth, and nose. The product was found to attract 93% of dust mites, kill 74% of dust mites within 48 h, and kill nearly 100% of dust mites within 72 h.

At present, Chinese people with allergic diseases account for approximately 30% of the total population (approximately 400 million), with allergies especially affecting those who are young [﻿28﻿]. Based on the prevalence of childhood asthma in 2010, there were 6.7 million children with asthma in China alone. Therefore, the potential consumer group for this product is very large. If the efficacy and safety of this mite bait product can be confirmed for allergic diseases via this test, the product will provide a new tool for the treatment of such allergic diseases in the future.

Additionally, the random grouping, double-blind, crossover test method adopted in this study reduces the influence of selection bias, measurement bias, and other errors on the test results. Each subject will receive two schemes successively, which allows before-and-after comparison, eliminating individual differences while allowing inter-group comparison.

However, due to the long period of this study, problems, such as loss to follow-up, exit, and decline in compliance, may easily occur. Ensuring that each case is in the same condition in each phase of treatment is difficult.

To improve study compliance, we will contact the parents or guardians through WeChat during the study and receive the questions raised by them in real time. Parents and guardians will give consent for the collection of indoor dust mite specimens by staffs who will receive training and provide assessment of the complete specimen collection in strict accordance with sampling procedures. In terms of trial safety, we will track all the events during the study until the incident is alleviated, the situation is stable, other explanations of the incident are obtained, or the contact with the subjects is lost. Subjects are able to drop out of the study at any time and continue to receive standard treatment for rhinitis and asthma in the outpatient department.

In summary, this study will scientifically and objectively evaluate the effect of mite bait on improvement of rhinitis and asthma symptoms, which might provide a convenient means for prevention and treatment of allergic diseases involving the airways in children.

## Trial status

Protocol version and date: April 25, 2019.

Start date: 28 July 2019

End date: 28 June 2020

## Data Availability

Data sharing is not applicable to this article, as no datasets are generated or analysed during the current study.

## Abbreviations

AR: Allergic rhinitis
HDMs: House dust mites
Th2 cells: T helper type 2 cells
VAS: Visual analogue scale
RCAT: Rhinitis Control Assessment Test
RQLQ: Rhinoconjunctivitis Quality of Life Questionnaire
ACQ-5: Asthma Control Questionnaire-5
